# Theoretical Study of the β-Cyclodextrin Inclusion Complex Formation of Eugenol in Water

**DOI:** 10.3390/molecules23040928

**Published:** 2018-04-17

**Authors:** Elena Alvira

**Affiliations:** Department of Physics, University of La Laguna, 38202 La Laguna, Tenerife, Spain; malvira@ull.edu.es; Tel.: +34-922-318-258

**Keywords:** cyclodextrins, eugenol, interaction energy, molecular mechanics, molecular dynamics, inclusion complex

## Abstract

The interaction between eugenol and β-cyclodextrin in the presence of water is studied by molecular mechanics and dynamics simulations. A force field model is used in molecular mechanics to determine the interaction energy and the complex configuration at the absolute minimum. The van der Waals term is the main contribution to the total energy, and so directly determines the configuration of the inclusion complex. The formation of inclusion complexes is simulated by molecular dynamics, in which their configurations are deduced from the position probability density that represents the preferred location and orientation of the guest in the simulation. When eugenol approaches from the rims of β-cyclodextrin, it tends to enter the cavity, remain inside for a short period and then exit from it. The guest tends to include the phenyl ring inside the cavity in the most probable configurations. Two inclusion complex configurations are proposed, each with the hydroxyl and methoxyl groups pointing towards one different rim of β-cyclodextrin. The initial guest orientation is the main factor determining these configurations. The model presented in this study reproduces the experimental findings on inclusion complex formation and proposes two possible complex configurations, one previously suggested by different authors.

## 1. Introduction

Cyclodextrins (CDs) are macrocyclic molecules composed of glucose units (six for α-CD, seven for β-CD, eight for γ-CD, etc.) forming truncated cone-shaped compounds. These give rise to cavities of different internal diameters, capable of containing molecules of different structure, size, and composition [1,2,3]. The ability of CDs and derivatized cyclodextrins to form inclusion complexes makes them useful in catalysis and chiral resolution of racemic compounds. Such processes are extensively employed in various research fields and technological applications. A well-known experimental outcome is that the size of the guest must be adequate to achieve maximum binding affinity in each CD, depending on molecular properties such as its composition and geometry [4,5,6,7]. There are certain general characteristics for the type of molecules capable of being included totally or partially inside the cavity of CDs, but each case must be analyzed individually. CDs and their inclusion complexes have been theoretically studied using several computational methods: molecular mechanics (MM) [8,9], molecular dynamics (MD) [6,10], and Monte Carlo simulations (MC) [11,12].

Eugenol (EG) is a phenol derivative that can be extracted from certain essential oils such as clove oil, basil, bay leaf, nutmeg, or cinnamon. It is used in the food, perfume, and pharmaceutical industries due to its ability to display biological activities such as antibacterial, antifungal, anesthetic, antiallergic, antioxidant, anticarcinogenic, antiinflammatory, and many other properties [13,14]. Instead of its multiple applications, EG presents some disadvantages such as light sensitivity or poor water solubility. The inclusion complex formation with CDs can increase its aqueous solubility and reduce the undesirable effects There is experimental evidence for eugenol inclusion complex formation in β-CD and some of its derivatives, both in solid state and aqueous solution [15,16,17]. These studies demonstrated that the molecular volume of EG fits the cavity size of β-CD, and that host and guest form inclusion complexes. They also suggested that the phenyl ring of EG is partially inside the cavity and within the hydroxyl and methoxyl groups that project outside the wider rim of β-CD. Among the studies related to EG, there are conformational studies of EG using Semiempirical and Density Functional Theory methods [18,19,20]. There are also theoretical studies of the inclusion complexes formed between water-soluble CD-grafted chitosan derivatives and EG, by means of molecular dynamics simulation [21]. In the inclusion complexes formed in this latter case, the guest also orients the hydroxyl and methoxyl groups towards the wider rim of the cavity. However, there are no previous molecular simulations of EG and β-CD with water.

The aim of the present study is to theoretically examine the interaction between EG and β-CD in the presence of water, based on molecular mechanics (MM) and molecular dynamics (MD) simulations. The MM simulation calculates the interaction energy between EG and β-CD and deduces their configuration at absolute minimum energy, but this interaction occurs in processes where the molecules are moving and cannot always reach such a configuration. The MD simulation studies the molecular movements due to their mutual interactions, this method being more appropriate to describe the process of forming complexes. The model attempts to reproduce the capacity of inclusion complex formation and establish the complex configuration. The method applied was previously used to determine the interaction energy and β-cyclodextrin inclusion complex formation of molecules with different size, shape, and compositions [22,23,24,25]. The interaction potential and simulation method used in this study are presented in Section 3. Section 2 evaluates the interaction energies between β-CD and EG, and discusses the main results of a molecular dynamics simulation regarding the formation of the inclusion complex. The results obtained are corroborated by the experimental findings.

## 2. Results and Discussion

### 2.1. Molecular Mechanics Simulation

**Figure 1:**
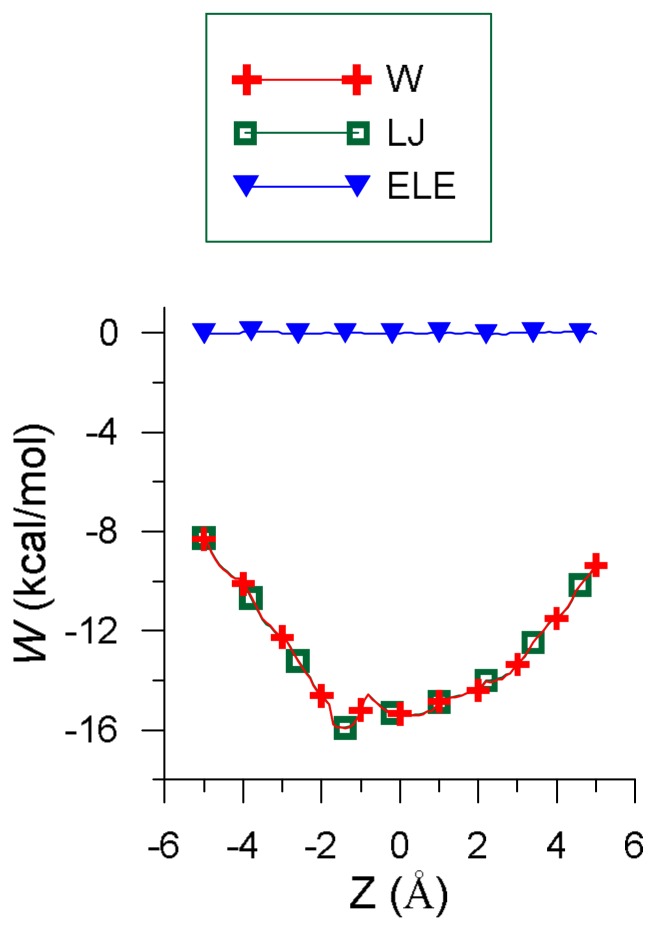
The variation in intermolecular energy ($E_{\mathrm{inter}}$) through the cavity (*W*) and its different contributions.

Figure 1 represents the penetration potential (*W)* along with its contributions. *W* resembles a well potential because the interaction energy is deeper inside than outside the cavity, which represents the force attracting EG into β-CD. The values of *W* and the van der Waals (LJ) term are nearly the same because the order of magnitude of the electrostatic energy (ELE) is 2 × 10^−2^ kcal/mol, and the H-bond term only contributes to $E_{\mathrm{inter}}$ at some positions of the guest outside the cavity. The small amount of electrostatic energy is due to the presence of water, whose dielectric constant (ε) is 80, although this contribution is similar for smaller values of ε. For instance, the electrostatic contribution for solvents like ethanol (ε = 26) is about 7 × 10^−2^ kcal/mol.

**Figure 2:**
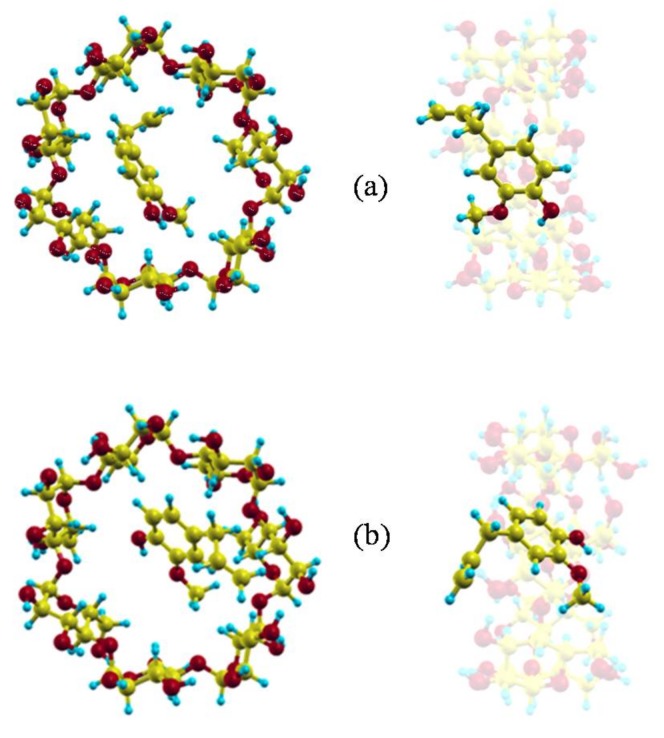
(**a**) The inclusion complex configuration of minimum energy and (**b**) another inclusion complex with similar energy but different configuration.

The minimum value of the interaction energy ($E_{\min}$) is −10.22 kcal/mol, greater than the minimum value of $E_{\mathrm{inter}}$ (−15.91 kcal/mol) because $E_{\mathrm{intra}}$ is positive (5.69 kcal/mol). The main contribution to $E_{\mathrm{intra}}$ is the torsional energy (3.98 kcal/mol), followed by the bond term (1.60 kcal/mol). Eugenol is located inside the cavity in the $E_{\min}$ configuration (inclusion complex configuration), parallel to the cavity axis, with its centre of mass near the narrower rim of β-CD and the O atoms pointing towards the wider rim (Figure 2a) [26]. The guest molecule is superimposed on the CD in Figure 2 for clarity. However, Figure 1 shows that the values of *W* near the cavity centre are similar to the minimum value of $E_{\mathrm{inter}}$, the differences being less than 1 kcal/mol. This means that EG can form β-CD inclusion complexes with similar energy but different configurations. In these complexes the guest centre of mass is located nearer the wide rim of β-CD and with different orientations, but its phenyl ring tends to stay within the cavity (Figure 2b) [26].

**Figure 3:**
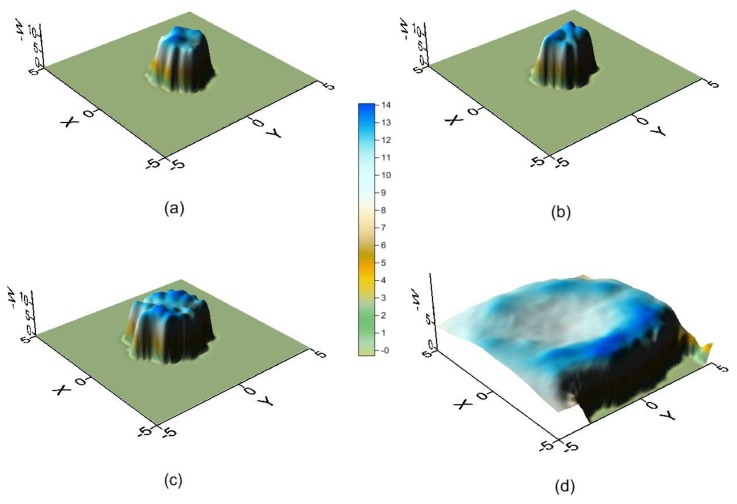
The potential energy surface (PES) near the narrower rim (**a**); centre (**b**,**c**); and wider rim of the cyclodextrin (CD) (**d**).

Figure 3 represents the potential energy surface (PES) near the narrower rim (Figure 3a), centre (Figure 3b,c) and wider rim of the CD (Figure 3d). The size of the regions where the energy is attractive increases with the diameter of the cavity, and near the wider rim the guest tends to locate its centre of mass outside the β-CD. The energy is lower around the cavity walls than near the centre of the host and is again seen to be similar in the regions (Figure 3b,c), although the absolute minimum energy is located in (Figure 3b).

### 2.2. Molecular Dynamics Simulation

**Figure 4:**
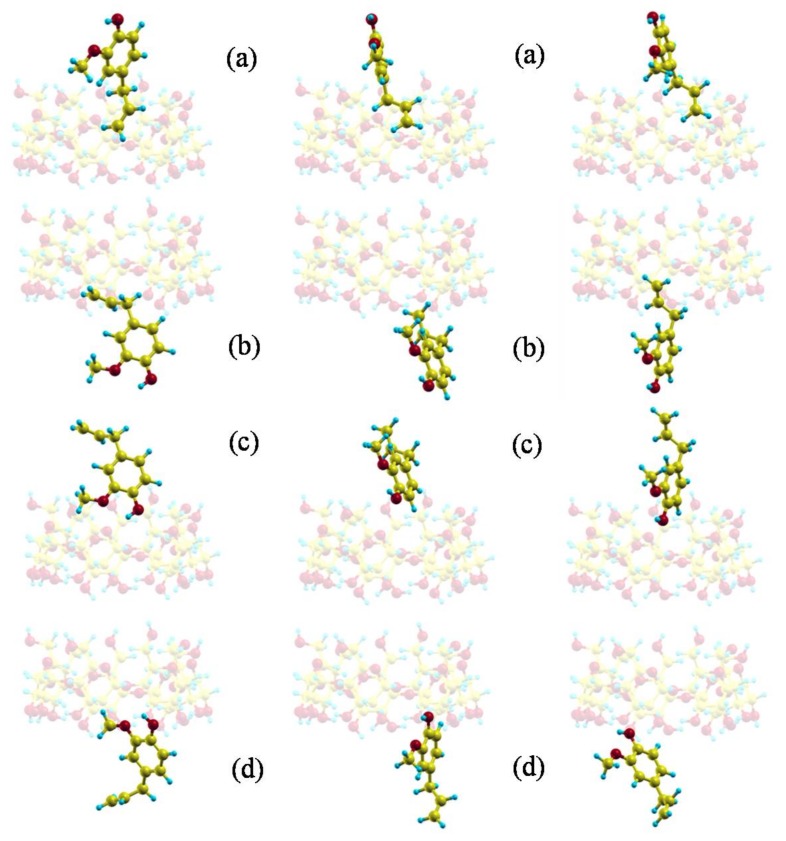
Starting dispositions of eugenol (EG) in the trajectories: (**a**) the guest centre of mass near the narrower rim of CD with the propene of EG pointing towards the cavity, (**b**) the guest centre of mass near the wider rim of CD with the propene of EG pointing towards the cavity, (**c**) the guest centre of mass near the narrower rim of CD with the phenyl ring of EG pointing towards the cavity, (**d**) the guest centre of mass near the wider rim of CD with the phenyl ring of EG pointing towards the cavity.

The movements of EG in the trajectories, and then the residence time *t*, interaction energy *E*, binding free energy *F* and position probability density depend on the initial values of the guest disposition and velocities. Whereas the velocities hardly influence the simulation, the initial centre of mass and orientation of EG determine the subsequent process. The initial dispositions of EG in the simulation are represented in Figure 4 [26], three trajectories in each relative position between host and guest. The results obtained in the simulation show that if the guest approaches the β-CD from the cavity rims, it tends to enter the cavity, remain inside for a short period (residence time *t*) and then exit from it, although not always passing through the cavity. When the guest is partially inside the cavity in the trajectories, it tends to include the phenyl ring because it thus adopts more stable configurations.

The mean value of the energy in each trajectory, and therefore in the simulation, depends on the movements of the guest. The value of $E_{mean}$ is −2.39 kcal/mol, the contribution of $E_{\mathrm{inter}}$ is −8.06 kcal/mol, and $E_{\mathrm{intra}}$ 5.67 kcal/mol. $E_{mean}$ is greater than $E_{\min}$ because the energy of every position with a probability other than zero contributes to the average energy in the trajectories. The van der Waals term contributes the most to $E_{\mathrm{inter}}$, and the torsional energy to $E_{\mathrm{intra}}$ in the simulation. However, these energies do not tell us if the complex formation is energetically favourable with respect to the reagents, whereas the binding free energy *F* does. *F* varies from −7.02 to −10.34 kcal/mol in the simulation, since $F_{mean}$ = −9.15 kcal/mol. The initial guest disposition influences $F_{mean}$ and $E_{mean}$, because they are due to movements of EG in the trajectories where the guest positions and orientations depend on the initial conditions.

**Figure 5:**
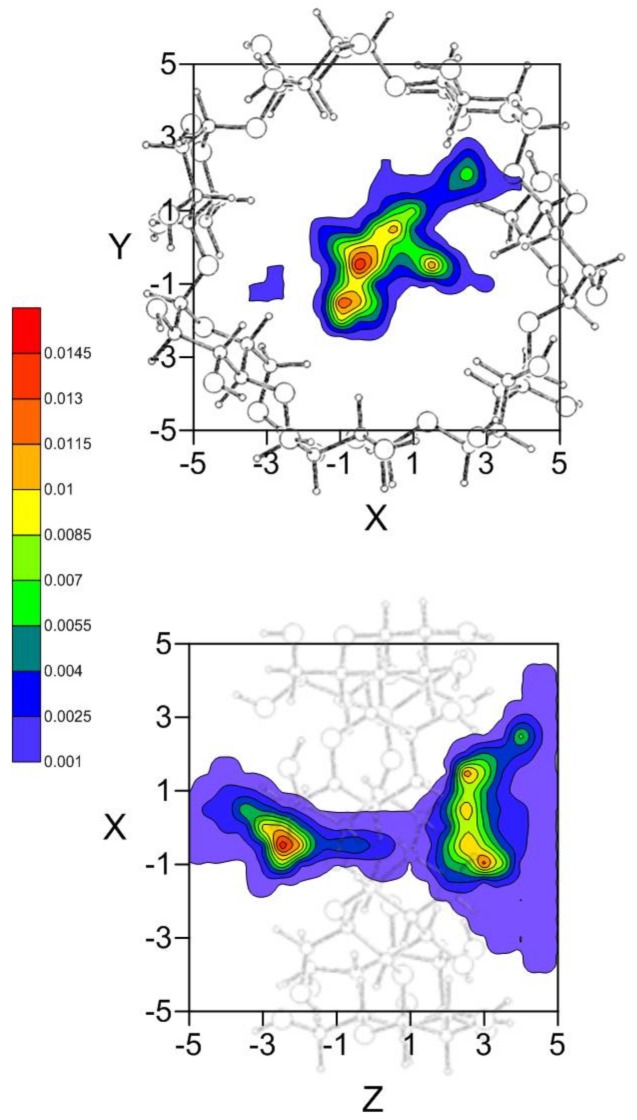
Projections in the XY and XZ planes of the position probability density for the inclusion of EG in β-CD. A schematic representation is included of the projections of β-CD in those planes for clarity.

As seen in Section 2.1, there are different inclusion complexes formed by β-CD and EG with similar interaction energies and different configurations; the phenyl ring of EG is always located inside the host, along the *Z*-axis. The preferred location of the guest in the MD simulation, and therefore the capacity to form inclusion complexes, is deduced from the position probability density (Figure 5). EG tends to locate its centre of mass preferably nearer either rim of β-CD, near the centre at the narrower rim (about 12.5%) but closer to the cavity wall at the wider rim (about 20%).

**Figure 6:**
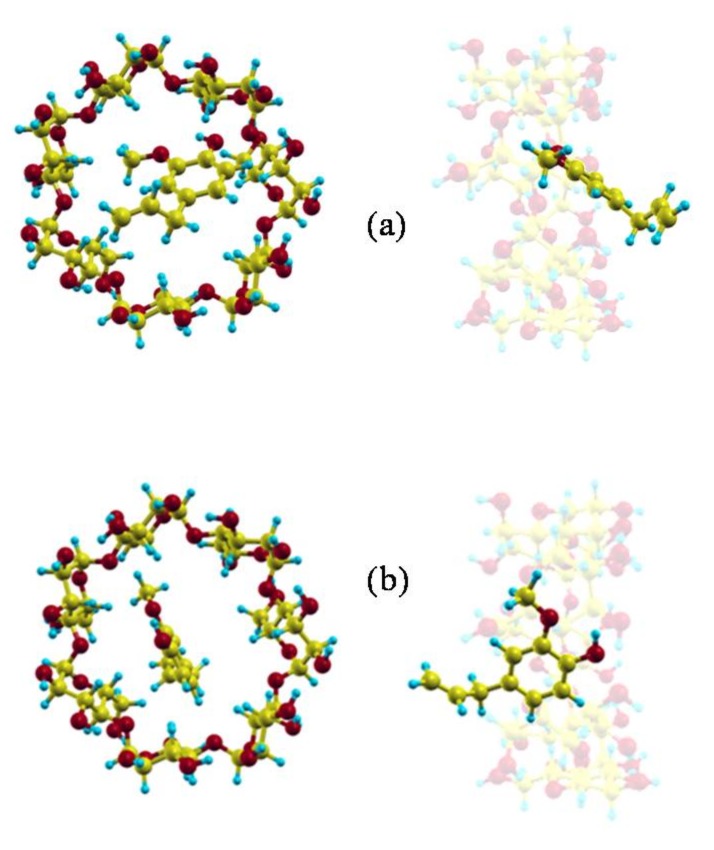
The most probable guest orientations corresponding to the preferred centre of mass positions: (**a**) near the wider rim of β-CD; (**b**) near the narrower rim of β-CD.

The guest orientation in these zones of highly probable presence does not remain constant, it varies continually according to the small energy differences between the complex configurations. However, the size of the guest does not permit a rotation of 180° with respect to the cavity axis inside the β-CD; this rotation only occurs outside the β-CD before entering the cavity. Once inside, the guest orientation is under strong restrictions due to the inner cavity size, and the variations are small. Some of the most probable guest orientations corresponding to the preferred centre of mass positions are shown in Figure 6 [26]. It can be concluded from the MD simulation that EG can form inclusion complexes with β-CD in which the phenyl ring is always inside the cavity, in agreement with the experimental findings. There are two types of inclusion complexes deduced from MD, whose configurations (Figure 6a,b) are very different because the hydroxyl and methoxyl groups are pointing towards either rim of β-CD. Nevertheless, none of these configurations agree with that of minimum energy, because the sizes of the host and guest do not allow the latter to move freely inside the cavity to adopt the minimum energy configuration. The inclusion complex in which the hydroxyl and methoxyl groups are projecting outside the wider rim of β-CD (Figure 6b) is that suggested by several other authors [15,16,17]. If these results are validated by experimental findings, it can also be concluded that there are other possible inclusion complex configurations.

**Figure 7:**
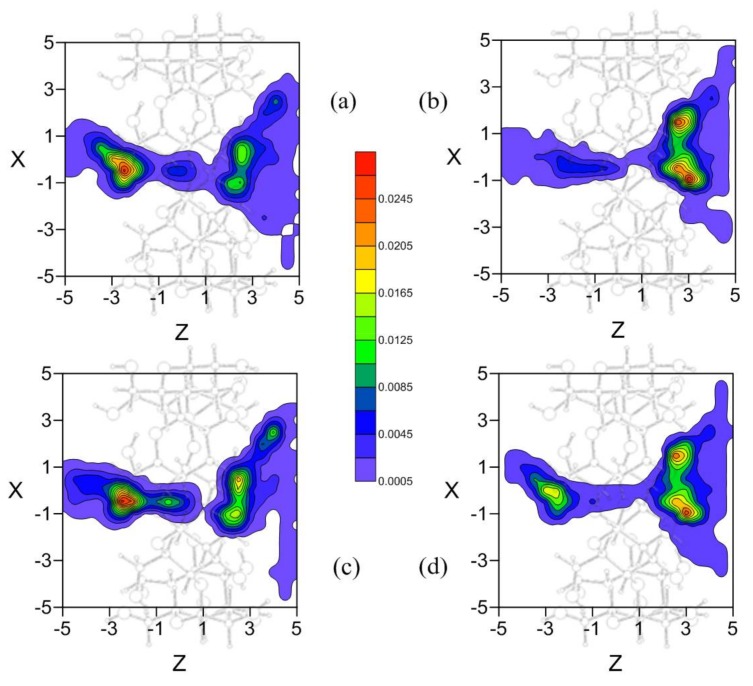
Projections in the XZ plane of the position probability densities for the inclusion of EG in β-CD calculated from trajectories with different initial dispositions: (**a**) the propene of EG projecting to the rims of β-CD; (**b**) the phenyl group of EG projecting to the rims of β-CD; (**c**) the initial centre of mass of EG near the narrower rim of β-CD; and (**d**) the initial centre of mass of EG near the wider rim of β-CD. A schematic representation is included of the projection of β-CD in this plane.

The two possibilities are obtained by considering the same number of trajectories in the MD with the mentioned initial relative positions, in order that all the different ways the molecules can approach each other appear in the simulation. To demonstrate that all the trajectories do not contribute equally to the more probable configurations, different position probability densities are determined (Figure 7). The density represented in Figure 7a corresponds to the trajectories in which the propene of EG is pointing towards the rims of β-CD, independently of the guest centre of mass (Figure 4a,b). In this case EG would form an inclusion complex like Figure 6b, after moving through the cavity with the initial orientation or changing it before entering β-CD. However, from the position probability density (Figure 7b) calculated with the trajectories in which the initial orientation of phenyl group of EG is towards the rims of β-CD (Figure 4c,d), the more probable inclusion complex is like Figure 6a. Therefore the initial guest orientation decisively influences the configuration and probability of forming one of this type of inclusion complexes. To assess the influence of the initial centre of mass of EG in the simulation, position probability densities are determined by considering the trajectories with initial positions near either rim of β-CD, independently of the guest orientation. If the guest approaches β-CD from the narrower rim (Figure 7c) or the wider rim (Figure 7d) it can reach both types of configurations, thus proving that the main factor determining inclusion complex formation is the initial guest orientation. This result also implies that establishing the inclusion complex configuration from experimental findings reveals the way host and guest preferably approach each other.

The initial guest disposition also influences the time during which the host-guest interaction is attractive enough for them to remain close to each other. The shorter residence times correspond to the trajectories with initial dispositions near the narrower rim of CD. Residence times in the simulation vary from 96 ps to 860 ps (t_mean_ = 360.3 ps), and the guest usually spends this residence time inside the cavity, as seen in the last paragraph.

## 3. Materials and Methods 

### 3.1. Molecular Mechanics Simulation

The driving forces contributing to the formation of complexes with CDs are due to the electrostatic, van der Waals, hydrophobic and H-bond interactions. Whereas the electrostatic, van der Waals and H-bond interactions are modelled by different analytical functions in molecular simulation methods, the hydrophobic interaction is one of the less understood. Traditionally, negative enthalpy and negative entropy changes observed in the experimental studies have been associated to a small contribution of the hydrophobic effect to CD complexation. However, investigations with supramolecular complexes recently show the importance of the replacement of high-energy water by guest molecules to the formation of complexes in cavities, although they show that this contribution is much smaller for flexible hosts like CDs than others cavities [27,28]. Modeling the process of this replacement would provide a method to theoretically take into account the hydrophobic effect in the interaction with CDs. However, the presence of water in the process is not represented in the present study by discrete solvent molecules, but a uniform continuous medium and thus the hydrophobic interaction cannot be included in the computational model. The interaction energy *E* between EG and β-CD is modelled by the sum of the intramolecular $E_{\mathrm{intra}}$ and intermolecular $E_{\mathrm{inter}}$ energies, as in the Assisted Model Building with Energy Refinement (AMBER) force field [29,30]. The intramolecular energy is modelled by a sum of the torsional energy, bond stretching and bending functions, and represents the conformational adaptation of the guest and host. The intermolecular energy is determined by a sum of the van der Waals (Lennard-Jones potential), electrostatic, and H-bond terms:

(1) $$E=\sum_{i<j}\left[\frac{A_{ij}}{R_{ij}^{12}}-\frac{B_{ij}}{R_{ij}^{6}}+\frac{q_{i}q_{j}}{\varepsilon R_{ij}}\right]+\sum_{H-bonds}\left[\frac{C_{ij}}{R_{ij}^{12}}-\frac{D_{ij}}{R_{ij}^{10}}\right]+{\sum_{bonds}k_{r}\left(r-r_{eq}\right)}^{2}+\;+\sum_{angles}k_{\theta }{\left(\theta -\theta_{eq}\right)}^{2}+\sum_{dihedrals}\frac{V_{n}}{2}\left[1+\cos\left(n\phi -\gamma \right)\right]$$

where *r* represents bond lengths, θ bond angles, ϕ torsional angles of molecules, and $R_{ij}$ the distance between the ith atom of the guest and the jth atom of β-CD The presence of water in the process is represented by a uniform continuous medium, with a dielectric constant ε = 80 in the electrostatic contribution to $E_{\mathrm{inter}}$. All atoms in the guest molecule are considered because some H atoms can contribute decisively to the formation of H-bonding between host and guest, and this may be reflected in the interaction energy *E*. The atomic coordinates of β-CD, its net atomic charges [31] and the AMBER force field parameters are taken from the literature [32,33]. The molecular configuration and atomic point charges of EG are calculated by the Hartree-Fock method using the 6-31G** basis set, implemented in the MOLPRO package [34,35]. The origin of the reference system is located at the centre of mass of the CD and the space-fixed frame over the principal axis of the β-CD, where the *Z* axis is collinear with the cone axis (thus the *XY* plane is parallel to the cone base). The configuration of EG is given by the coordinates of its centre of mass and the molecular orientation, defined by the Euler angles formed with respect to the absolute frame (*X*, *Y*, *Z*). The method is the same previously applied to study the interaction between β-CD and various molecules, therefore the energy *E* is calculated for different positions and orientations of the guest centre of mass, inside and outside the CD [22,23,24,25]. The complex configuration of EG with β-CD in water is determined from MM as the position and orientation of the guest in the absolute minimum energy $E_{\min}$. To obtain this minimum value, a grid is defined (−5 ≤ *X* ≤ 5, −5 ≤ *Y* ≤ 5, −5 ≤ *Z* ≤ 5) in which the distance between two consecutive points is 0.1 Å. At each grid point, *E* is determined for different orientations (about 23,000) and the minimum value is assigned to each location. The results obtained from the simulation are shown by the penetration potential (*W*), potential energy surface (PES), complex configuration, the minimum value of *E* ($E_{\min}$) and its different contributions. The penetration potential *W* is the curve joining the minimum intermolecular energy for every plane Z = constant, and represents the variation in $E_{\mathrm{inter}}$ through the cavity. The capacity of EG to form a β-CD inclusion complex in water is deduced from the MM simulation by the configuration of $E_{\min}$ (the complex configuration), and it is considered an inclusion complex if the guest is totally or partially located inside β-CD. The PES is calculated at each grid point from the average Boltzmann energy corresponding to different guest orientations, instead of the lowest energy [36,37], because EG does not always reach the minimum energy orientation whilst moving inside and around the CD. The PES is represented by partitioning the range of *Z*-axis variation in the β-CD into four parts, depending on the position of the guest molecule’s centre of mass near the narrower rim, centre or wider rim of the cavity. The length of each domain is about 2.5 Å and the potential surface area for each region is determined as the minimum value of the average Boltzmann energy, for every point on the plane in the corresponding interval of the *Z*-axis.

### 3.2. Molecular Dynamics Simulation

The classical equations of motion for the molecules are solved in MD to obtain the trajectories of EG due to its interaction with β-CD. A basic result of classical mechanics is that the translational motion of the molecule’s centre of mass is governed by the total force acting on the body, whereas the rotation about the centre of mass depends on the total applied torque. The total force on the molecule is determined as the sum of the forces acting on each of its atoms (${\vec{f}}_{i}=-{\vec{\nabla }}_{{\vec{r}}_{i}}E$). In order to avoid the problem of divergence in the orientational equations of motion, four quaternion parameters have been used as generalized coordinates. The trajectories are determined with different initial values of the guest disposition (centre of mass and orientation) and velocities (translational and rotational). The magnitude of the initial velocities depends on the temperature of the process (293 K), but their direction as well as the initial orientation of EG in each trajectory are determined randomly. When the initial guest centre of mass is located outside the CD near the cavity walls, it does not enter the CD, but rather continues moving around the host, tending to move away. When the starting position of EG in the simulation is located near the cavity rim, it tends to enter the cavity and remain inside for a short period, forming a stable complex (residence time *t*). It then moves away from the CD, as previously found in the MD simulation of different molecules with β-CD [22,23,24,25]. Basically, there are four relative positions between the molecules: the guest centre of mass near either rim of CD, with one end of it (the phenyl ring or the radical) pointing towards the cavity. Twelve trajectories are calculated in the present study, three starting from each relative position between the molecules. In this way, the contributions of different initial guest dispositions are considered equally in the simulation. Moreover, we determine some trajectories with the same initial orientation but different starting centres of mass of EG (near either cavity rim), so as to analyse separately the influence of these factors on the simulation. The length of each trajectory is not defined by the simulation time or the number of steps, we stopped integrating the equations of motion when the guest was located outside the β-CD, in positions where its interaction with the CD was not attractive enough to re-enter cavity [25]. The configuration, and kinetic and potential energies, were registered every 100 steps of 1 fs. We used an in-house program written in Fortran, and the equations of motion to perform constant-temperature molecular dynamics were integrated numerically using a variant of the leap-frog scheme (proposed by Brown and Clarke) [38], constraining the rotational and translational kinetic energies separately [39].

The results obtained for each trajectory were: the interaction energy *E* and its different terms, the binding free energy *F*, residence time *t* and position probability density. The average values of *E*, its different terms, *F* and *t* obtained for the simulation were also determined ($E_{mean}$, $F_{mean}$, $t_{mean}$). Whereas the residence time represented the time during which the interaction between β-CD and EG is attractive enough to remain close to each other (inside or outside the cavity), the capacity to form inclusion complexes was deduced from the position probability density, which represented the preferred location of the guest in the simulation. A guest molecule is able to form an inclusion complex with β-CD when it has greater probability to remain totally or partially inside the cavity. This position probability density was calculated by dividing the number density in a volume element by the total amount of possible centre of mass positions for the guest. The number densities of presence or number of guest positions in each volume element was defined by a grid [36,37]. There are several generalized Born Models to calculate electrostatic binding free energies [40], but the electrostatic contribution to $E_{\mathrm{inter}}$ is nearly constant inside the cavity (Figure 1) and thus in the trajectories. Moreover the van der Waals term is about $10^{3}$ times greater than the electrostatic potential energy, making the process of inclusion complex formation essentially dependent on this contribution. Therefore, the total energy of the complex during the trajectories was considered to determine the binding free energy *F* in the simulation using:

(2) $$F=-k_{B}T\ln\left(\sum_{i}\exp\left(-W_{i}/k_{B}T\right)\right)$$

where $W_{i}$ is the energy of the complex during the trajectories, T the temperature of the process (293 K) and $k_{B}$ Boltzmann’s constant [29]. 

## 4. Conclusions

A molecular mechanics simulation of the interaction between eugenol and β-cyclodextrin in water was presented in this study. The van der Waals term is the main contributor to the total energy, particularly inside the cavity, and so directly determines the configuration of the inclusion complex. The small electrostatic contribution to the total energy is due to the presence of water, whereas the intramolecular energy reflects the structural relaxation of the host and guest molecules. The molecular mechanics simulation of the interaction between EG and β-CD in the presence of water demonstrates the capacity of EG to be included in β-CD, forming stable complexes in which the hydroxyl and methoxyl groups are pointing towards the wider rim of the cavity.

The process of forming inclusion complexes was simulated by MD, showing that when EG approaches from the rims of β-CD, it tends to enter the cavity, remain briefly inside then exit from it, spending in the process a mean time of about 360.03 ps. The guest is not static inside β-CD, it varies in both its centre of mass position and orientation, although the latter is restricted by the molecular size. The guest is partially inside the cavity in the most probable configurations, although it always tends to include the phenyl ring. Two types of configurations are proposed for the inclusion complexes, each with the hydroxyl and methoxyl groups pointing towards a different rim of β-CD. The model presented in this study reproduces the capacity of eugenol to form inclusion complexes with β-CD, in agreement with experimental findings. It proposes two possible configurations of the complex, one of them suggested previously by several authors. This indicates that the main factor influencing the type of inclusion complex formed is the initial guest orientation.
